# An outbreak of acute respiratory disease caused by a virus associated RNA II gene mutation strain of human adenovirus 7 in China, 2015

**DOI:** 10.1371/journal.pone.0172519

**Published:** 2017-02-22

**Authors:** Xiaoxia Yang, Qiongshu Wang, Beibei Liang, Fuli Wu, Hao Li, Hongbo Liu, Chunyu Sheng, Qiuxia Ma, Chaojie Yang, Jing Xie, Peng Li, Leili Jia, Ligui Wang, Xinying Du, Shaofu Qiu, Hongbin Song

**Affiliations:** 1 Institute of Disease Control and Prevention, Academy of Military Medical Sciences, Beijing, China; 2 Deprtment of Infection Control, Wuhan General Hospital of Guangzhou Military Command, Wuhan, China; University of Hong Kong, HONG KONG

## Abstract

Human adenovirus 7 (HAdV-7) strains are a major cause of acute respiratory disease (ARD) among adults and children, associated with fatal pneumonia. An ARD outbreak caused by HAdV-7 that involved 739 college students was reported in this article. To better understand the underlying cause of this large-scale epidemic, virus strains were isolated from infected patients and sequence variations of the whole genome sequence were detected. Evolutionary trees and alignment results indicated that the major capsid protein genes hexon and fibre were strongly conserved among serotype 7 strains in China at that time. Instead, the HAdV-7 strains presented three thymine deletions in the virus associated RNA (VA RNA) II terminal region. We also found that the mutation might lead to increased mRNA expression of an adjacent gene, L1 52/55K, and thus promoted faster growth. These findings suggest that sequence variation of VA RNA II gene was a potential cause of such a severe HAdV-7 infection and this gene should be a new-emerging factor to be monitored for better understanding of HAdV-7 infection.

## Introduction

Human adenoviruses (HAdVs) are pathogenic viruses that can usually cause acute respiratory disease (ARD), acute follicular conjunctivitis, haemorrhagic cystitis, gastroenteritis, myocarditis, meningoencephalitis, and even death [1]. Outbreaks of adenovirus infection had been reported worldwide and are recognized as the leading cause of febrile illness and respiratory diseases [2]. Based on serology, genome sequencing, and biological characteristics, HAdVs have been classified into seven species (HAdV-A to G), which include more than 64 serotypes [3]. Different genotypes have been associated with distinct clinical symptoms. For instance, respiratory diseases have been linked mostly to HAdV-B serotypes 3, 7, and 14, and HAdV-C serotypes 1, 2, 5, and 6 [4, 5]. Among these serotypes, HAdV-7 is frequently associated with severe respiratory disease [6]. Numerous reports have covered the consequences of HAdV-7-induced epidemics among children and in crowded spaces. These include febrile pharyngitis and pneumonia accompanied by acute respiratory disease, some of which have been associated with fatal outcomes [7].

The severity of HAdV-7 infection depends on many factors, such as virus virulence, viral transmission environment, or physical condition of the host, and dictates whether occurrence is sporadic *vs*. epidemic, or mild *vs*. severe [8–10]. Genetic alterations are the leading cause of changes in the virulence of HAdV-7, as can be seen in the new emerging HAdV-7 strains with genomic variants such as SmaI mutation and VA RNA gene deletions, and could increase the severity of illnesses [11, 12]. A better understanding of viral genetic changes will improve the prediction and prevention of future ARD outbreaks caused by HAdV-7.

Traditionally, capsid hexon and fibre genes have been used to detect genomic variation, as they also determine the HAdV serotypes. However, according to recent reports, the hexon and fibre genes of HAdV-7 were remarkably conserved across time and space [13]. With the application of whole genome sequencing, two HAdV-7 strains identified in separate ARD outbreaks contained a 12-bp deletion in the VA RNA II gene, even though hexon, fibre, and other genes were highly conserved [12]. Nevertheless, there is no clear evidence whether the VA RNA II gene mutations contributed to past HAdV-7 outbreaks.

Here, we report a large-scale outbreak of severe acute respiratory disease associated with HAdV-7 that occurred in Wuhan, China, in 2015. To assess whether this outbreak was related to genetic variation of the VA RNA gene of the incriminated HAdV-7 strain, we analysed the whole genome sequence variation and viral growth of the Wuhan isolates. Knowledge of a link between VA RNA II genetic variation and HAdV-7 virulence may help us understand the function of this gene and take effective measures to control HAdV-7 prevalence.

## Materials and methods

### Outbreak investigation

An outbreak of ARD was reported in a college in Wuhan city, Hubei Province, between the 20^th^ of January and 21^st^ of February, 2015. Health officials, medical staff, epidemiologists, and laboratory technicians were designated as an epidemic prevention team to investigate and control the outbreak. The names, onset times, clinical symptoms and informed consent for sample collection and subsequent research usage of the students were documented by trained medical staff and epidemiologists. Copies of the case records of the hospitalized students were provided by the corresponding doctors. All the protocols used in these investigations were approved by the Institutional Review Boards of the Centre of Disease Control and Prevention of China, and the Ethics Committee of Wuhan General Hospital.

### Sample collection, detection, and serotyping

Nasopharyngeal swabs of the patients were collected using 5-mL collection tubes (Yocon, China). Pure Link Viral RNA/DNA Mini Kits (Invitrogen, Carlsbad, CA, USA) were used to extract nucleic acids. Suspected pathogens, such as adenovirus, coronavirus, parainfluenza virus, influenza A virus, influenza B virus, respiratory syncytial virus A, *Bordetella pertussis*, *Legionella pneumophila*, *Chlamydophila pneumoniae*, *Streptococcus pneumoniae*, *Haemophilus influenzae*, and *Mycoplasma pneumoniae* were detected with Seeplex^®^RV12 ACE and Seeplex^®^ Pneumabacter detection kits (Seegene, Seoul, Korea). After confirmation of HAdV infection, real-time PCR primers for HAdV serotypes 7, 11, 14, and 55 were used for serotyping (S1 Table).

### Virus isolation and gene sequence analysis

The samples of nasopharyngeal swabs were inoculated into A549 cells cultured in DMEM. The viruses were isolated by repeated freeze-thaw and preserved in -80°C. Primer sequences used for the amplification of hexon, fibre and VA RNA genes have been published elsewhere [14]. The whole genome was sequenced using Dideoxyterminator Sanger sequence analysis and primers used were listed in S2 Table. The sequences were aligned with MUSCLE within MEGA6.05 software. Neighbour-joining methods were adopted to construct phylogenetic trees and 1000 bootstrap replicates were used to evaluate their topological accuracy.

### Detection of L1 52/55K mRNA

Viral RNA was extracted using an Ultrapure RNA extraction kit (DNase I included, CW0597; KangWei Biotech, China). The primers used for the detection of L1 52/55K mRNA were F: 5’-TGCAAAGAGCTCTAACGGGG-3’ and R: 5’-CTTCCAAGTACTCGCCCTCC-3’. Reference genes VA RNA II and E1 were detected using primers VA-F: 5’-GAGCCAGTGCTGCGTCAA-3’, VA-R: 5’- TTAGAAACGTCGCGGAAA-3’; and E1-F: 5’-CCACCTACGCTGCACGAT-3’, E1-R: 5’- ACCTCTGCCGCTTTCCAC-3’, respectively. KAPA SYBR FAST qPCR Kits (KK4601; Kapa Biosystems, Woburn, MA, USA) were used for amplification.

### Virus growth curve

Viruses (1 × 10^2^ genome copies) were propagated in human A549 cells cultured in 12-well plates and were collected 6, 9, 12, 18, 21, 24, 36, 48, and 72 h after infection. The cultures were subjected to repeated freezing and thawing thrice and centrifuged at 12000×g for 10 min. Then, 200 μl of supernatants were used for DNA extraction using Pure Link Viral DNA Mini Kits and virus titres were determined by detecting the number of viral E1 gene copies by real-time PCR. The E1 gene fragment was cloned into the T vector (pEASY-T1 Simple Cloning Kit, CT111-02; TransGen Biotech, Beijing, China) and diluted to 0.5 × 10^2^–0.5 × 10^9^ copies/μL as standard samples.

### Statistical analysis

All experiments were performed at least three times. The data are presented as means ± standard error of the mean (SEM) and were analysed using Student’s *t*-test. Two-tailed *P*-values < 0.05 were considered statistically significant.

## Results

### Epidemiological and clinical characteristics

**Fig 1:**
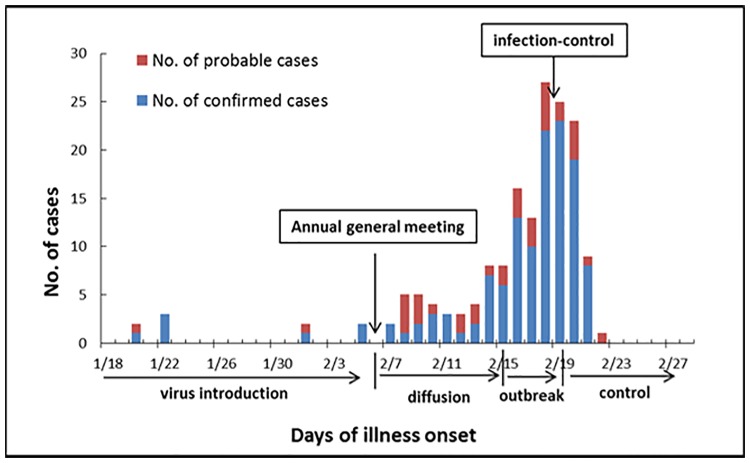
Number of ARD patients admitted to hospital and confirmed cases of HAdV-7 infection in this outbreak.

In 2015, an outbreak of acute respiratory disease occurred in a college in Wuhan, China. Of 4113 students, 828 (20.1%) developed the illness and 169 of them were admitted to Wuhan General Hospital between the 20^th^ of January and 21^st^ of February, 2015 (Fig 1). The average age of the hospitalized students was 19 years (between 17 and 24 years). In this study, we took the 169 hospitalized cases for subsequent epidemiological and clinical investigation. The development of the outbreak unfolded as follows. From late January to early February, a few sporadic cases were hospitalized. Then, after an annual college general meeting on the 6^th^ of February, the virus began to spread and led to an outbreak. Control measures, such as isolation, disinfection, and medication, were introduced on the 19^th^ of February. From that point, the number of new infections decreased gradually and after the 23^rd^ of February, no additional patients required hospitalization.

According to case reports, patients presented typical respiratory infection symptoms, such as fever (92.3%), cough (83.4%), sore throat (62.1%), swelling of tonsils (47.9%), and a runny nose (26.0%). Among them, 92 patients (54.4%) were diagnosed with upper respiratory infection, 77 (45.6%) with pneumonia (Table 1), and 121 (71.6%) presented fever above 39°C. In addition, nearly half (n = 37) of the patients suffering from swollen tonsils (n = 81) presented severely enlarged tonsils. Finally, there were also 17 cases of acute bilateral pneumonia.

**Table 1:** Characteristics of HAdV infected patients.

Characteristic		Value (n = 169)
Clinical symptom		
Cough		141 (83.4%)
Sore throat		105 (62.1%)
Expectoration		104 (61.5%)
Headache		48 (28.4%)
Weak		65 (38.5%)
Pharyngeal lymphoid follicular hyperplasia		11 (6.5%)
Purulent exudate on the tonsil		9 (5.3%)
Running nose		44 (26.0%)
Myalgia		45 (26.6%)
Diarrhoea		18 (10.7%)
Fever	40–41°C	26 (15.4%)
	39–39.9°C	95 (56.2%)
	38–38.9°C	30 (17.8%)
	<38°C	5 (3.0%)
	Total	156 (92.3%)
Body function indexes		
WBC count (10^9^ cells/litre)	>10	34 (20.1%)
	<4	19 (11.2%)
Lymphocyte % (>0.4%)		168 (99.4%)
Neutrophil % (>0.7%)		169 (100%)
Bradycardia (<60 times/min)		4 (2.4%)
Tachycardia (>100 times/min)		52 (30.8%)
Tachypnea (>20 times/min)		6 (3.6%)
Diagnosis		
Upper respiratory infection		92 (54.4%)
Pneumonia		77 (45.6%)
Severe cases (admission to intensive care units)		8 (4.7%)

### Virus detection and analysis of viral gene sequences

Of the 4113 students, 739 were identified as HAdV-7-positive by real-time PCR. The ratio among hospitalized patients was 76.9% (130 out of 169). Twenty viral strains were isolated from hospitalized positive patients and cultured in the laboratory; after which hexon, fibre, and VA RNA genes were amplified and sequenced. Alignments of these genes of the isolated 20 virus produced 100% similarity.

**Fig 2:**
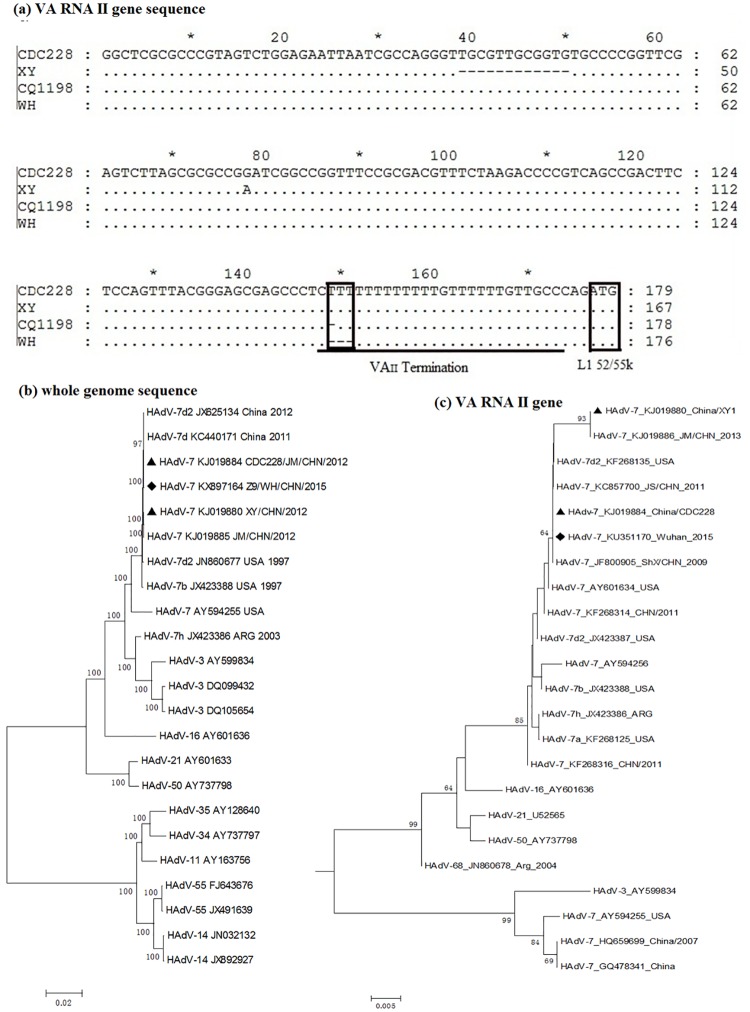
Alignment and phylogenetic analysis of VA RNA II gene and whole genome. (a) Alignment of VA RNA II gene sequences from different HAdV-7 strains. The empty black box in the VAII region indicates the sites of missing T. The empty black box to the right indicates the start codon of the L1 52/55K gene. Phylogenetic tree corresponding to HAdV-7 (b) whole genome and (c) VA RNA II gene sequences is presented. The Wuhan strain reported in this article is marked by (♦), whereas the reference strains CDC228 and XY1 are indicated by (▲). The scale bar indicates units of nucleotide substitutions per site.

Next, we used the BLASTn tool to find the strains with closest relation to the sequenced hexon, fibre, and VA RNA genes. Both hexon and fibre genes showed 100% identity with the HAdV-7 isolate CQ1198 (GenBank accession number JX625134), which belongs to the HAdV-7d type [10]. The VA RNA gene (GenBank accession number KU351170) also showed the highest identity to this HAdV-7 strain, but it lacked two thymines (T). A comparison of the Wuhan strains with strains CDC228 (KJ019884) and XY1 (KJ019880) previously isolated by our laboratory [12], revealed that the former had three fewer T in the VAII terminal region (Fig 2).

Then, we chose the Z9/WH strain (GenBank No. KX897164) of these Wuhan isolates as representative to make whole-genome sequencing, and the comparison result with strain CDC228 revealed that the Z9/WH strain had some other but insignificant mutations except for VA RNA gene (Table 2).

**Table 2:** Comparative genomic analysis of the outbreak Z9/WH strain with strain CDC228.

Region	Location(CDC228)	Gene	Nucleotide Change (amino acid substitution)	
			CDC228	Z9/WH
E1B	1631	20 kDa small T antigen	G(W)	C(C)
E2B	5069	DNA polymerase	T^a^	C
	7842		T(243^rd^E)	G(A)
	7882		G(230^th^L)	C(V)
	8057		G	A
	8081		G	A
L1	10748	VAII	TTT	–^b^
L2	15453	non-coding region	AAAAAA	–
E3	28601	20.3 kDa glycoprotein	A(I)	G(V)

^a^The bases without brackets denote silent mutations.

^b^“–” denotes deletion mutation.

### Phylogenetic tree construction and analysis

Phylogenetic trees based on hexon, fibre, and VA RNA gene sequences were constructed respectively. As all sequences were identical, one sample Wuhan strain (KU351170) was taken as representative. And the Z9/WH strain (GenBank No. KX897164) was chosen for the whole-genome sequence and DNA polymerase gene analysis (Figs A and B in S1 Appendix).

According to the phylogenetic trees, the hexon and fibre genes of the Wuhan strains clustered with the HAdV-7d2 strain CQ1198 (JX625134) and other HAdV-7 strains found in China in recent years. The DNA polymerase gene and whole genome sequence of HAdV-7 found in China in recent years were located within the same branch but with a little difference. And the VA RNA I sequences from all serotype 7 strains clustered together and the sequences were highly conserved except for occasional mutations in the termination region (Figs B and C in S1 Appendix). In contrast, the VA RNA II genes were quite variable even within the same serotype (Fig 2).

### Viral L1 52/55K gene expression

**Fig 3:**
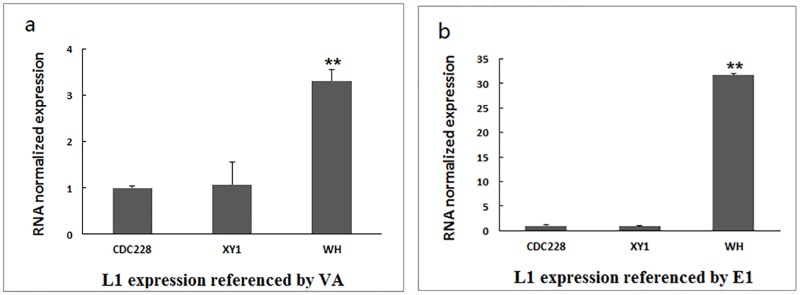
L1 52/55K mRNA expression assay using real-time PCR. L1 52/55K mRNA expression against (a) VA RNA, n = 3, mean ± SEM, **P = 0.0039 *vs*. CDC228; and (b) E1, n = 3, mean ± SEM, **P = 0.0098 *vs*. CDC228. CDC228, XY1, and WH correspond to KJ019884, KJ019880, and Wuhan strains, respectively.

We analysed the expression of the viral gene L1 52/55K using VA RNA and E1 as reference genes. Compared with strains CDC228 and XY1, the representative Wuhan strain displayed substantially higher L1 52/55K expression. L1 52/55K gene expression did not differ between CDC228 and XY1 strains (Fig 3), even though the latter had 12 fewer bases in the VA RNA region than the former [12].

### Growth kinetics of viral replication

**Fig 4:**
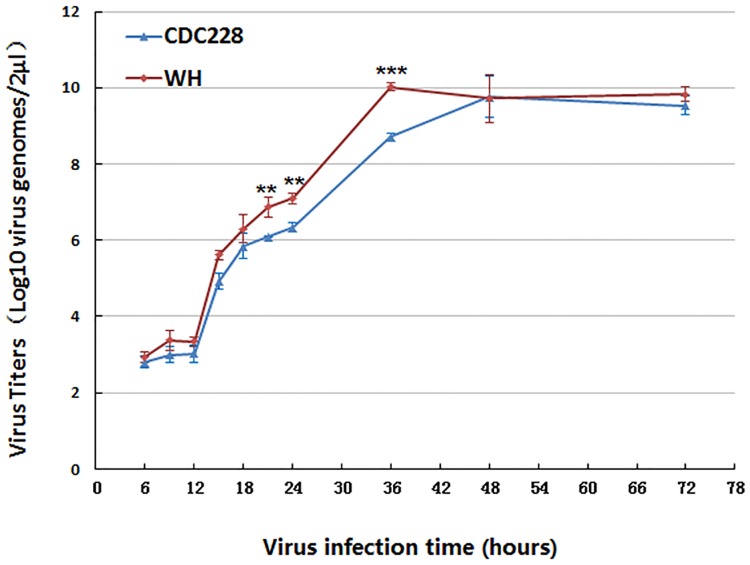
Growth kinetics of Wuhan and CDC228 strains. Virus titres were determined in human A549 cells cultured in 12-well plates by detecting virus genomes using real-time PCR (n = 3, mean ± SEM, **P ≤ 0.01, ***P ≤ 0.001 *vs*. CDC228).

Finally, we compared viral replication kinetics between the CDC228 strain and one of the Wuhan strains (Fig 4). The latter displayed faster growth than CDC228, which contains a complete VA RNA II terminal region.

## Discussion

Different HAdV serotypes have been shown to be prevalent in different geographic areas [15–18], and different types of HAdV infection have been related to various levels of severity [19–22]. HAdV-B types (HAdV-3 and HAdV-7) can cause respiratory tract infection and result in severe pneumonia and death [7, 23]. Under special conditions, such as fatigue and crowding, HAdV-7 can cause outbreak of acute respiratory disease [6, 24].

Here, we report an outbreak of ARD caused by HAdV-7 among young college students. According to clinical characteristics, this outbreak brought 169 inpatients with poor conditions, like fever above 39°C (71.6%), pneumonia (45.6%) and 8 ICU (intensive care unit) cases. To understand the reason behind this large scale outbreak, genomic variations were detected in our article.

Hexon and fibre capsid proteins are the main antigenic determinants of HAdV serotypes. In a previous work on two other HAdV-7 outbreaks, we found that most genes had few or no mutations besides those in VA RNA genes, which displayed a 12-bp deletion [12]. Therefore, here, we analysed the sequences of hexon, fibre, and VA RNA genes primarily. The hexon, fibre, and VA RNA I genes clustered quite intensely within serotype 7 in the phylogenetic trees, suggesting the conserved prevalence of HAdV-7 in China in recent years. However, the VA RNA II gene had undergone several mutations, even within the same serotype. Besides, the whole genome sequence analysis barely showed any extra mutations, except for two amino acids substitutions in the DNA polymerase gene, which were not located within the functional regions of DNA polymerase [25]. Therefore, more attention should be paid to the VA RNA II gene for future monitoring of HAdV-7 infection.

How the lost T in the VA RNA II terminal region played influence on HAdV-7 is still unknown. Interestingly, the HAdV VA RNA genes are located right in front of the L1 52/55K gene [26], which plays an important role in forming mature, infectious virions [27, 28]. In fact, we show that the start codon of L1 52/55K mRNA is located only two nucleotides after the terminal region of VA RNA II (Fig 2). We first tried to measure the expression ratio between the VA RNA II and L1 52/55K gene of different HAdV-7 strains. However, given the possibility that the mutation in the VA RNA II gene might alter the expression of the gene itself, we chose instead to assess the expression of L1 52/55K against that of E1A. Being the first expressed early gene [29], E1A could reflect the expression of HAdV genes and avoid being influenced by late genes. We show that L1 52/55K mRNA was up-regulated compared with strains CDC228 and XY1, confirming the possibility that deletion in the terminator of VAII could exert an effect on this adjacent gene. The terminal region of VA RNAs is characterized by a cluster of T surrounded by a GC-rich stretch [30]. This region is transcribed by RNA polymerase III [31], which is prone to misreading the termination sequence and read through the next genes [30]. Accordingly, the loss of T in the terminator might destabilize the secondary structure of the terminal stem and increase the chance of continued transcription of L1 52/55K by RNA polymerase III. Considering the role of L1 52/55K protein in promoting the formation of infectious virions, the up-regulation of L1 52/55K might explain the rapid growth of the Wuhan strain and this outbreak. In addition, a previous report suggested that the L1 52/55K gene, which recombined from HAdV-B16, was a reason for the virulence of HAdV-7 strains predominated in China [32]. The WH strain identified in this outbreak shared 100% identity with this reported L1 52/55K gene. Thus, the up-regulated expression of this L1 52/55K gene could lead to increased virulence of WH strain.

In summary, we report here an uncommon outbreak of HAdV-7 among college students in Wuhan, China, in 2015. Sequence analysis revealed several missing T in the VA RNA II terminal region. This mutation, in turn, caused up-regulation of an adjacent gene, L1 52/55K. The latter promoted faster growth of the HAdV-7 strain, thus providing a causal link between the VA RNA II gene and a severe clinical outcome. Taking into account the phylogenetic results on the conserved nature of HAdV-7 hexon and fibre genes in China in recent years, VA RNA genes represent a new determinant factor for HAdV-7 virulence upon infection. However, further work, such as functional verification of the missing T bases by constructing a deletion mutant of strain CDC228 or reverse mutant of the WH strain, should be done to elucidate the function of the VA RNA II gene and its relationship to the increased growth capacity of the HAdV-7 strain.

## Supporting information

S1 Appendix Fig A. Phylogenetic analysis of HAdV-7 hexon and fibre genes. Fig B. Phylogenetic analysis of DNA polymerase gene and VA RNA I gene of HAdV-7. Fig C. Sequence alignment of HAdV-7 VA genes. Fig D. Sequence alignment of VA II gene of HAdVs.
(DOCX)

S1 Table Primers used for HAdV serotyping. (DOCX)

S2 Table Primers used for HAdV whole genome sequencing. (DOCX)
