# Supplementary material for: An outbreak of acute respiratory disease caused by a virus associated RNA II gene mutation strain of human adenovirus 7 in China, 2015
Source: PLoS One. 2017 Feb 22;12(2):e0172519. doi: 10.1371/journal.pone.0172519 (PMC5321423; doi:10.1371/journal.pone.0172519)

**Fig A**. **Phylogenetic analysis of HAdV-7 hexon and fibre genes.** Phylogenetic trees corresponding to HAdV-7 (a) hexon and (b) fibre gene sequences are presented. The Wuhan strain reported in this article is marked by (♦), whereas the reference strains CDC228 and XY1 are indicated by (▲). The scale bar indicates units of nucleotide substitutions per site.


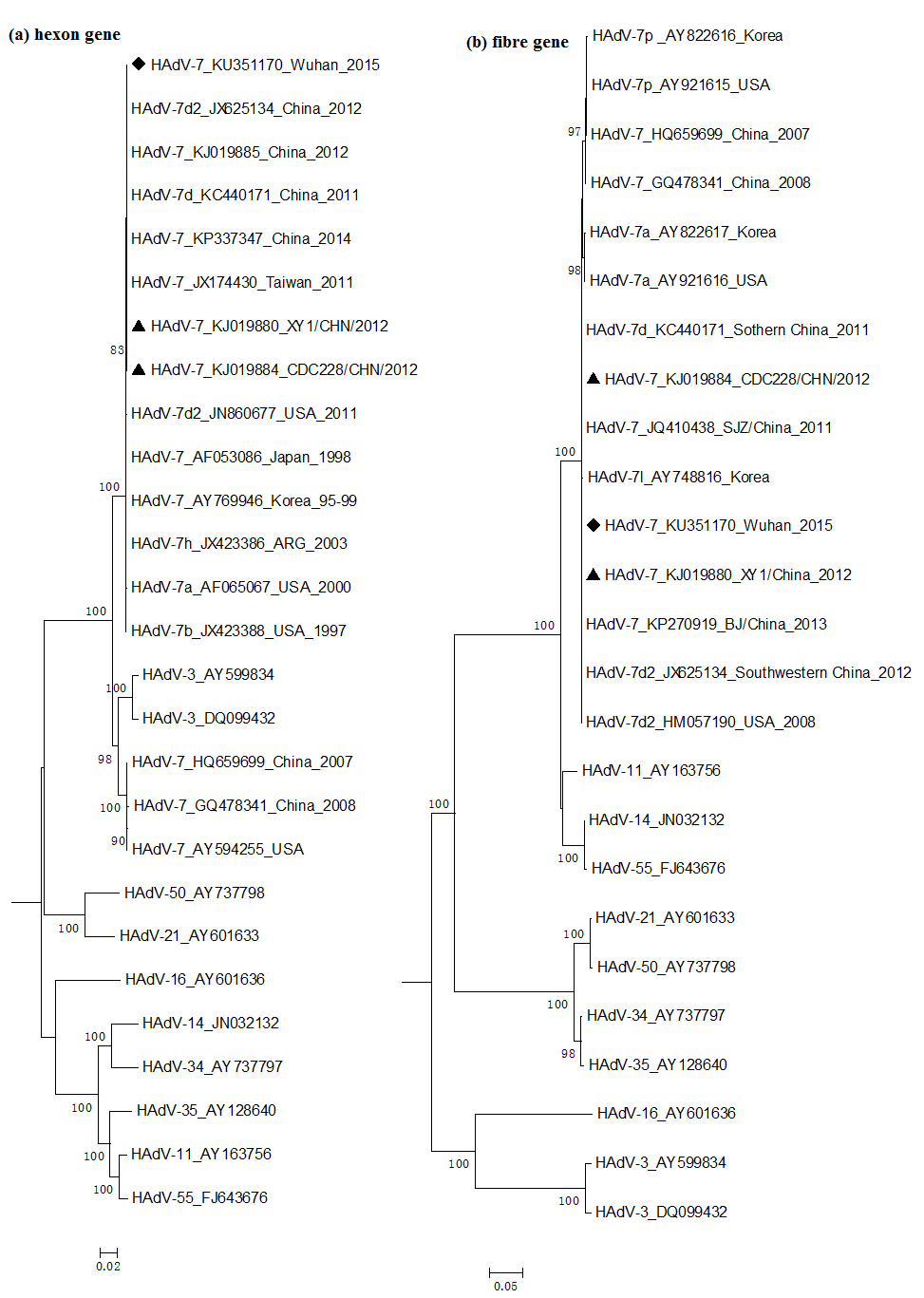


**Fig B**. **Phylogenetic analysis of DNA polymerase gene and VA RNA I gene of HAdV-7.** Phylogenetic trees corresponding to HAdV-7 (a) DNA polymerase gene and (b) VA RNA I gene.


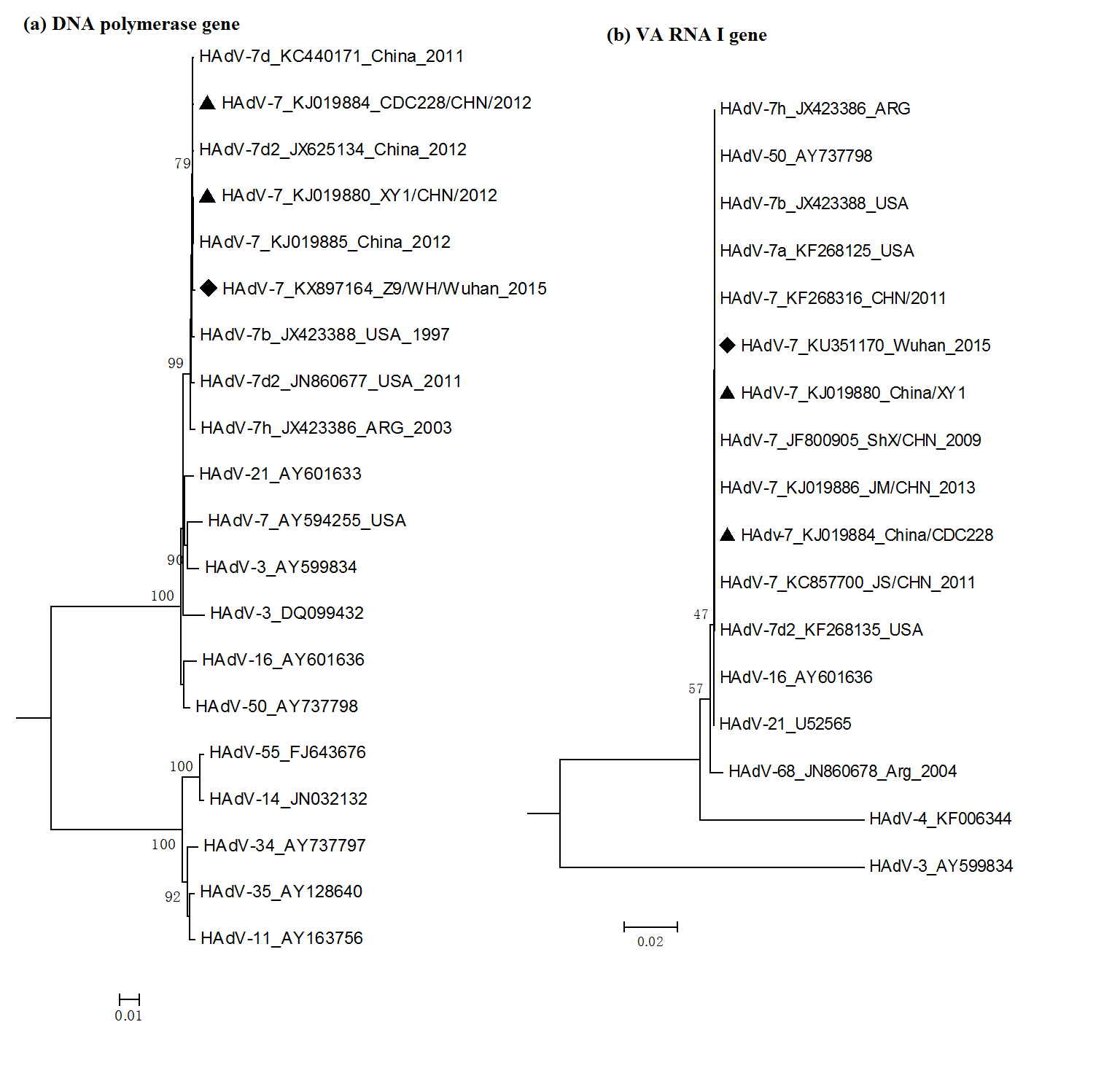


**Fig C. Sequence alignment of HAdV-7 VA genes.** Alignment of (a) VAI and (b) VAII gene sequences from HAdV serotype 7 strains. The empty black box represents the site of missing T.


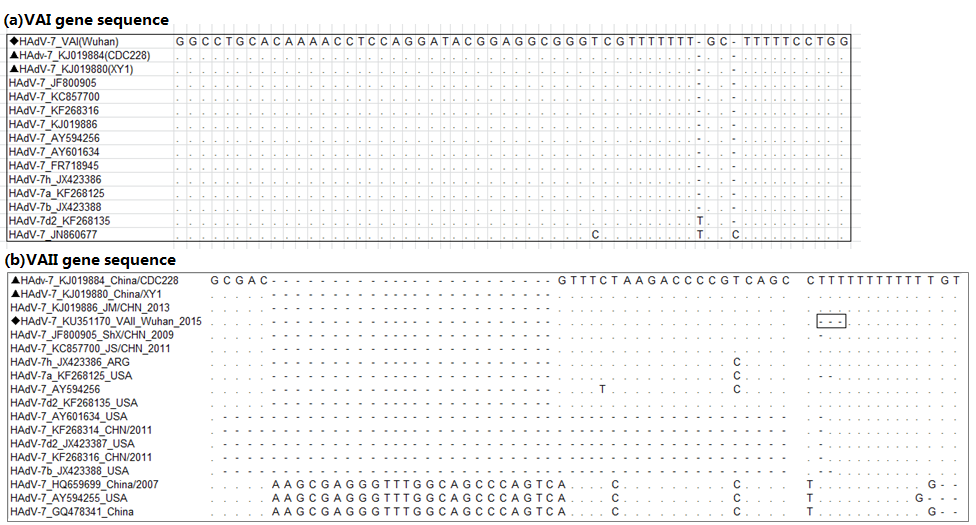


**Fig D. Sequence alignment of VA II gene of HAdVs.** Different nucleotides were showed in gray background.


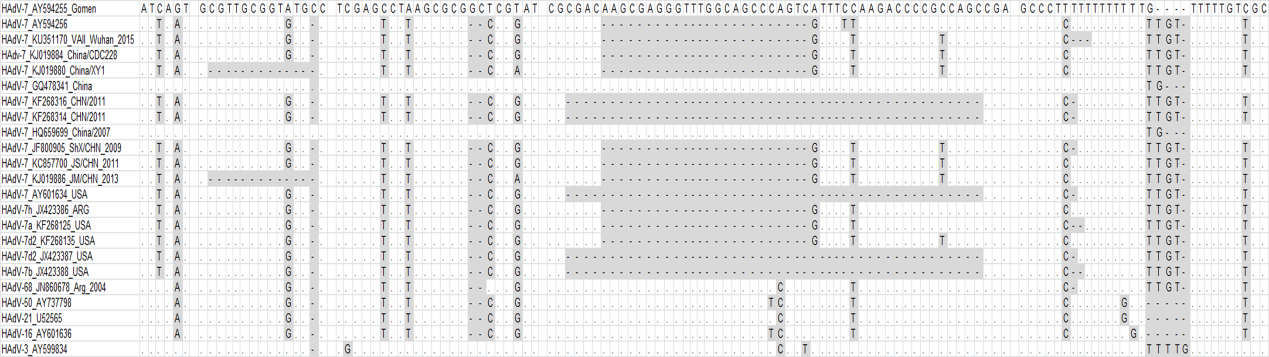

Supplement: S1 Appendix — Fig A. Phylogenetic analysis of HAdV-7 hexon and fibre genes. Fig B. Phylogenetic analysis of DNA polymerase gene and VA RNA I gene of HAdV-7. Fig C. Sequence alignment of HAdV-7 VA genes. Fig D. Sequence alignment of VA II gene of HAdVs. (DOCX) [file pone.0172519.s001.docx]
