# Supplementary material for: An outbreak of acute respiratory disease caused by a virus associated RNA II gene mutation strain of human adenovirus 7 in China, 2015
Source: PLoS One. 2017 Feb 22;12(2):e0172519. doi: 10.1371/journal.pone.0172519 (PMC5321423; doi:10.1371/journal.pone.0172519)
Supplement: S1 Table — (DOCX) [file pone.0172519.s002.docx]

**S1 Table. Primers used for HAdV serotyping.** HAdV-B strains were the main pathogens responsible for the respiratory infection, therefore primers based on HAdV-B type sequences were used to confirm HAdV infection. Serotypes 7, 11, 14, and 55 of HAdV-B were the common respiratory pathogens, thus primers of these serotypes were used for serotyping[^33^](#_ENREF_33).

|  | **Primer** | **Sequence(5'-3')** |
| --- | --- | --- |
| **HAdV-B** | qHAdV-UniF | TTTGAGGTYGAYCCCATGGA |
|  | qHAdV-UniR | AGAASGGTGTRCGCAGGTA |
|  | qHAdV-UniProbe | FAM-ACCACGTCGAARACTTCGAA-BHQ1 |
| **HAdV-7** | qHAdV7-F | GAGGAGCCAGATATTGATATGGAATT |
|  | qHAdV7-R | AATTGACATTTTCCGTGTAAAGCA |
|  | qHAdV7-Probe | FAM-AAGCTGCTGACGCTTTTTCGCCTGA-BHQ1 |
| **HAdV-11/55** | qHAdV11/55-F | CGGAGCAGCCAAATCAGAA |
|  | qHAdV11/55-R | CATGAGTGTCTGGAGTTTCCAAAT |
|  | qHAdV11/55-Probe | FAM-TGCGGCATCACAGAAAACAAACTTAAGTC-BHQ1 |
| **HAdV-14** | qHAdV14-F | GAAAATCATGGTGTGGAAGATGAA |
|  | qHAdV14-R | CAAGCTTGGTCTCCATTTAACTGA |
|  | qHAdV14-Probe | FAM-ACGGCATCGGTCCGCGAACA-BHQ1 |
