# Supplementary material for: An outbreak of acute respiratory disease caused by a virus associated RNA II gene mutation strain of human adenovirus 7 in China, 2015
Source: PLoS One. 2017 Feb 22;12(2):e0172519. doi: 10.1371/journal.pone.0172519 (PMC5321423; doi:10.1371/journal.pone.0172519)
Supplement: S2 Table — (DOCX) [file pone.0172519.s003.docx]

**S2 Table. Primers used for HAdV whole genome sequencing.**

| **Primers ID** | **F** | **R** | **size** | **start** | **end** |
| --- | --- | --- | --- | --- | --- |
| 1 | 5’-TGCGGTAAATGTGACGTAAA-3’ | 5’-TACAGGAATGGGCTTGCATA-3’ | 1181 | 106 | 1267 |
| 2 | 5’-GAGAATGCACTGCCACTTTA-3’ | 5’-TGCCACCAAACAGTTTCAGG-3’ | 1170 | 1066 | 2216 |
| 3 | 5’-GGTGCTTACTAGGTCTACGA-3’ | 5’-CCGTCAGATAAGGGCTAAAT-3’ | 1367 | 2101 | 3448 |
| 4 | 5’-GTGAGTAGTGGGGCAAGATG-3’ | 5’-CTGGGAAATGCAAATATGTG-3’ | 1267 | 3324 | 4571 |
| 5 | 5’-GGGGGTCTGAAACATCATAG-3’ | 5’-CGTGGCCTACACTTACAAGC-3’ | 1339 | 4445 | 5764 |
| 6 | 5’-CCCAGCCCACTCTGATACAA-3’ | 5’-AGCCAACCGCGTTATGACAT-3’ | 1269 | 5615 | 6864 |
| 7 | 5’-ATGGCATGGGAATTTGAAGA-3’ | 5’-GCATGACCACCATCGAGACA-3’ | 1357 | 6696 | 8033 |
| 8 | 5’-CAGTACTCGCAGCGATTCAC-3’ | 5’-CCGAACTCATCCGTCTTCTG-3’ | 1200 | 7890 | 9070 |
| 9 | 5’-CATAGGCGCTGGAAAAGGTA-3’ | 5’-CCACCGCCACACTTTCTCAT-3’ | 1220 | 8882 | 10082 |
| 10 | 5’-CCCCGGTGTTGATTGTGTAG-3’ | 5’-GGCTGATCCCTGTCACTTCA-3’ | 1241 | 9930 | 11151 |
| 11 | 5’-CCCGCCTATGATCTGGAGTT-3’ | 5’-TATACCAGCCCGGCCTCATC-3’ | 1349 | 10900 | 12229 |
| 12 | 5’-CGAAGCCTTTAGACAGCAAC-3’ | 5’-TGGCCTCAACAAAGAGTCAT-3’ | 1366 | 12090 | 13436 |
| 13 | 5’-AAGGGGGCGATAGAAGACAC-3’ | 5’-TGCTAAGTCGGCTTTCAGTA-3’ | 1353 | 13259 | 14592 |
| 14 | 5’-GCCTCCCGGTTATACAGTTT-3’ | 5’-TGCTAAGTCGGCTTTCAGTA-3’ | 1382 | 13230 | 14592 |
| 15 | 5’-AAAATGGAGTGCTGGAAAGT-3’ | 5’-GATGTAGGCGCAGTAGGAGT-3’ | 1259 | 14435 | 15674 |
| 16 | 5’-TAATACCGGTTGGGGACTGT-3’ | 5’-CCGTACTTTCGTCGTGATCT-3’ | 1383 | 15487 | 16850 |
| 17 | 5’-GATATCCAGCCTGAGGTCAA-3’ | 5’-GTGGGCATTCCGAGCTTTAC-3’ | 1296 | 16655 | 17931 |
| 18 | 5’-TATTAACGGCGTAGTGGATT-3’ | 5’-CTCCTTCGGTTGGTGTTACT-3’ | 1335 | 17726 | 19041 |
| 19 | 5’-ATCAGCCAGAGCCTCAAGTT-3’ | 5’-GAGTTGAAGCGGTGTTGTGA-3’ | 1287 | 18878 | 20145 |
| 20 | 5’-CCCAATGGACAATGTGAATC-3’ | 5’-CTCTGACCACGTCGAAGACT-3’ | 1202 | 19857 | 21039 |
| 21 | 5’-ACCCAGCCAATTATCCATAC-3’ | 5’-CCGGTCCCAACTGTGATTTC-3’ | 1298 | 20801 | 22079 |
| 22 | 5’-CATGCACTAAGGCTCTCGTT-3’ | 5’-AGAAGCCCAGAAAGACCAAG-3’ | 1336 | 21843 | 23159 |
| 23 | 5’-CTTCTCCCAGGCAGATACCA-3’ | 5’-ATGCCATTCTCTCCCTGTTG-3’ | 1328 | 22940 | 24248 |
| 24 | 5’-TTTACTGTCCCCGAAGTGCT-3’ | 5’-CCCGGTGTCAGTTTCAGACT-3’ | 1376 | 24041 | 25397 |
| 25 | 5’-TGGCCAACTACCTCTCCTAC-3’ | 5’-AGGCGTGGGAATCTCTTTAC-3’ | 1438 | 25194 | 16612 |
| 26 | 5’-ACCAGAAAACCAGCAGTTAG-3’ | 5’-ATGCAGGCGAGAGTCTATTC-3’ | 1229 | 26245 | 27454 |
| 27 | 5’-GTGGATGGCTATGATTGATG-3’ | 5’-CAGTGACAATGTTGGCAATA-3’ | 1307 | 27274 | 28561 |
| 28 | 5’-CCTCCCAGCAAAGAGAACAT-3’ | 5’-CCCACTACCACGGCAGTAAT-3’ | 1203 | 28362 | 29545 |
| 29 | 5’-ACGGAAGAGACTTGACCATA-3’ | 5’-TAATGGCAGGAGGTAGTGAA-3’ | 1281 | 29282 | 30543 |
| 30 | 5’-ATCTGATTTCCCCACATACT-3’ | 5’-AGGCCATTATTTGACAGTTG-3’ | 1307 | 30333 | 31620 |
| 31 | 5’-CCACCACACCACTCGTTAAG-3’ | 5’-TGAAATCAGAATGCGTTGCT-3’ | 1218 | 31442 | 32640 |
| 32 | 5’-GGGAATCATAATCCGAAAAC-3’ | 5’-CGCGATCTACAATTTGAAGT-3’ | 1201 | 32472 | 33653 |
| 33 | 5’-AATGCTGGCTTCAGTTGTAA-3’ | 5’-TGGGAAAATGACGTTGTAAG-3’ | 1579 | 33474 | 35035 |
